# The future of udder health: Antimicrobial stewardship and alternative therapy of bovine mastitis

**DOI:** 10.3168/jdsc.2025-0839

**Published:** 2025-09-25

**Authors:** Pamela L. Ruegg

**Affiliations:** Department of Large Animal Clinical Sciences, College of Veterinary Medicine, Michigan State University, East Lansing, MI 48824

## Abstract

Although antimicrobial usage is highly restricted in dairy cows, concerns about development and dissemination of resistant bacteria have led to a reexamination of mastitis therapies. In the past, almost all cases of clinical mastitis were treated symptomatically using intramammary antibiotics, and in many regions, most cows received intramammary antibiotic treatments at dry-off. On modern dairy farms, changes in the distribution of etiological agents have reduced the need to give antimicrobials for all mastitis cases. Culture-guided selective treatment protocols for nonsevere clinical mastitis have been adopted on many farms and align with principles of antimicrobial stewardship. Although some antimicrobial therapy is needed to maintain udder health, antimicrobial therapy is not effective or not necessary for resolution of many cases of mastitis. As the use of antimicrobials has declined, many alternative treatments have been explored, but few non-antimicrobial treatments have documented efficacy. The purpose of this paper is to review the concepts of antimicrobial stewardship on dairy farms from the US perspective, with an emphasis on current and future management strategies for udder health.

## Highlights

• Many farms can reduce dependence on the use of antimicrobials for mastitis treatment.
• Improved stewardship includes the use of culture-guided selective treatment protocols and reduced duration of antimicrobial treatments.
• Improved stewardship includes the use of selective dry cow therapy when possible.
• There is little evidence to support the efficacy of most non-antimicrobial treatments.
• Research to define in vivo efficacy of alternative therapies for mastitis is needed.

## Graphical Abstract

**Figure:**
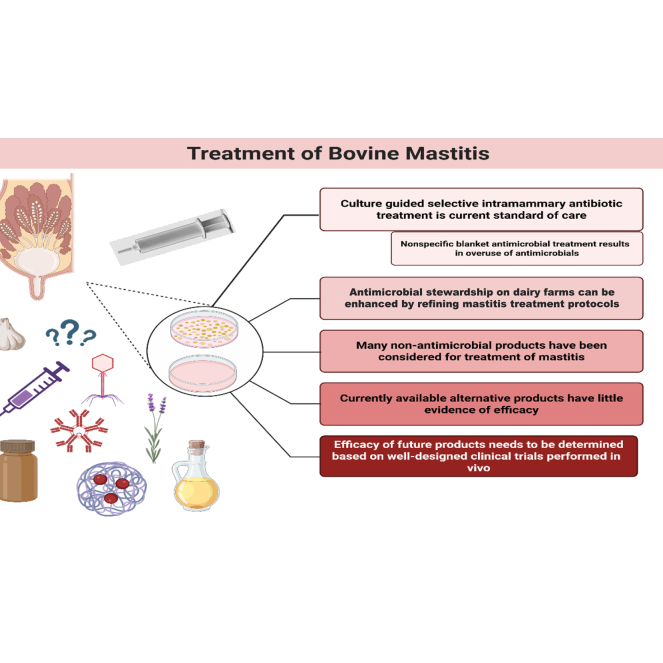
**Summary:** Mastitis is usually caused by bacterial infections of the udder, and most cases are currently treated using intramammary antimicrobials. Producers can reduce antimicrobial usage by adopting pathogen- and cowspecific selective treatment protocols. Several non-antimicrobial treatments are available, and researchers have described additional treatment options. Most alternative treatments lack efficacy data. Well-designed clinical trials performed in cows with naturally occurring mastitis are needed to determine the efficacy of alternative products. Graphical abstract created in BioRender (https://BioRender.com/t11r742).

In veterinary medicine, antimicrobial stewardship (**AMS**) is defined as systematic, proactive actions taken by veterinarians to preserve effectiveness and availability of antimicrobials (AVMA, 2018). On dairy farms, most antimicrobials are used to treat and prevent mastitis (Leite de Campos et al., 2021), so AMS programs must emphasize evidence-based treatment protocols for mastitis (Ruegg, 2022). Mastitis is defined as inflammation of the mammary gland and almost always occurs due to IMI, but many cases do not require antibiotic therapy during lactation. An understanding of the etiologies, the expected effectiveness of the immunological response to IMI and the costs associated with treatment are required to be able to assess the necessity and cost-effectiveness of therapy. Most IMI are caused by bacteria, although algae, yeasts, and other pathogens (including some strains of highly pathogenic avian influenza) have been associated with mastitis. After IMI is established, a nonspecific immune response results in inflammation that is used to detect and characterize the presentation. Subclinical mastitis (**SCM**) occurs when IMI results in an inflammatory response but the milk and udder remain normal in appearance. Detection of SCM is based on measuring the influx of neutrophils and is usually defined when the SCC of milk from individual quarters exceeds 200,000 cells/mL. At the herd level, bulk tank SCC is often used as an indicator of SCM, and values for US herds indicate considerable progress in controlling SCM. From 2000 to 2024, average bulk tank SCC declined from 326,000 to 181,000 cells/mL (CDCB, 2025), and based on monthly SCC values of cows tested by DHIA, the average prevalence of SCM in US herds has declined to ∼15%. However, the prevalence of SCM often increases with parity, and at the end of lactation, approximately half of cows in parity 3+ may have SCC values indicative of SCM. If the bulk tank SCC remains acceptable to the processer (usually <400,000 cells/mL), milk from cows with SCM is legal to sell. To limit financial losses due to discarded milk after treatment and to enhance bacteriological clearance, most treatments of SCM are performed at dry-off using intramammary (**IMM**) antimicrobials.

Clinical mastitis (**CM**) is detected by observation of visible changes in milk, with or without changes in the udder and cow. Abnormal milk is the only clinical sign for ∼50% of CM cases, whereas ∼35% also have inflamed udders and 5% to 15% of cows with CM are systemically ill (Ruegg, 2025). Clinical mastitis is the most frequently recorded disease of dairy cows and occurs in ∼20% to 40% of lactations, with greater incidence in multiparous cows (USDA, 2016). Milk that appears abnormal cannot be sold for human consumption (NCIMS, 2023) and must be discarded (or fed to calves), so it has limited or no value. Historically most CM cases were treated nonspecifically using IMM antimicrobials in the hope that treatment would hasten return to saleable milk, although recent research does not support that theory (Ruegg, 2025).

As producers have adopted effective preventive practices, the distribution of etiologies has changed, and many cases are caused by pathogens that are spontaneously cured or are outside the spectrum of activity of approved antimicrobials (Table 1). In many regions mastitis caused by *Streptococcus agalactiae* is rare and in some regions relatively few IMI are caused by *Staphylococcus aureus*. When developing treatment protocols, it is important to recognize that the distribution of etiologies vary between CM and SCM. Culture-negative milk samples account for ∼25% to 35% of CM cases. Most culture-negative cases of CM have achieved spontaneous bacteriological clearance before detection and have an excellent prognosis without antimicrobial therapy (Fuenzalida and Ruegg, 2019b). Although many milk samples obtained from quarters with SCM (high SCC) are also culture-negative, the failure to culture bacteria from those quarters does not always indicate that they are healthy. Chronically increased SCC in culture-negative quarters can occur because the inflammatory response has reduced the number of bacteria to below laboratory detection limits (usually 100 cfu/mL), and these IMI are often caused by pathogens that are nonresponsive to therapy. When bacteriological clearance occurs (either spontaneously or due to effective treatment), the SCC of affected quarters will gradually return to <200,000 cells/mL, but the time required to return to normal varies with etiology and may exceed 4 to 6 wk (Ruegg, 2025). In contrast, with or without bacteriological clearance, milk from cows with CM usually returns to a normal appearance within 3 to 5 d after detection of the case as the inflammatory process resolves. Thus, it is difficult for producers to discern the effectiveness of treatments, as clinical signs and SCC values can be misleading.

**Table 1:** Distribution of etiologies for clinical and subclinical mastitis

Item	Oliveira et al., 2013	Levison et al., 2016	Fredebeul-Krein et al., 2022	Rowe et al., 2020	McDougall et al., 2022	Niemi, et al., 2025
Location	Wisconsin, United States	Ontario, Canada	Germany	United States	New Zealand	Finland
Herds (n)	50	59	65	7^1^	44^2^	12^3^
Case type	Clinical	Clinical	Clinical	Subclinical	Subclinical	Subclinical
Cases (n)	622	936	854	1,078^4^, ^5^	704^4^	260
No growth (%)	27.3	19.6	20.3	74.6^6^	33	75.1
Coliform	33.1	11.5	22.2	2.3	<1	1.5
Streptococci and Strep-like organisms^7^ (%)	13.6	14.6	29.9	8.4	18.8	21.5
*Staphylococcus aureus*(%)	2.8	7.9	4.8	1.0	37.6	8.9
NAS and corynebacteria (%)	6.1	21.7	9.1	74.1	30.8	68.0
Other organisms and mixed (%)	17.1	24.7	13.7	14.2	12.8	0

1 All quarters of 1,275 cows cultured 2 d before dry-off.

2 Only California Mastitis Test–positive quarters from cows with SCC >500,000 cells/mL were cultured.

3 Quarters of cows with last test-day SCC >100,000 cells/mL were PCR tested 0 to 4 d before dry-off.

4 Culture-positive quarters only.

5 Culture-positive quarters enrolled.

6 Of 5,100 quarters cultured.

7 Includes non-*agalactiae Streptococcus* spp., *Lactococcus* spp., and *Enterococcus* spp.

A large variety of organisms have been identified from milk of cows with clinical and subclinical mastitis (Kurban et al., 2022). Among culture-positive cases on farms in North America, gram-negative bacteria are commonly recovered from CM, whereas most SCM cases are caused by gram-positive pathogens (Table 1). Recognition of this difference is vital because culture-guided, selective treatment protocols for CM emphasize use of antimicrobials primarily for treatment of cases caused by gram-positive pathogens (de Jong et al., 2023). At dry-off, the low prevalence of SCM caused by gram-negative pathogens (Rowe et al., 2020) indicates that narrow-spectrum IMM antimicrobials are appropriate when dry cow antimicrobial therapy is used. For both CM and SCM, principles of AMS suggest that use of narrow-spectrum IMM antimicrobials that target gram-positive bacteria are appropriate for routine treatment protocols and the broad-spectrum antimicrobials and injectable antimicrobials should be used only for selected cases with defined etiologies (Nobrega et al., 2020).

In most developed countries, antimicrobial usage (**AMU**) on dairy farms is highly restricted due to concerns about food safety and in order to reduce the potential dissemination of resistant bacteria and genes that may pose a risk to public health. Fewer antimicrobial classes and routes are approved for treatment of mature dairy cows, as compared with other food animals. Based on concerns about antimicrobial resistance, some regions have highly restricted use of some antimicrobials (Kuipers et al., 2016; Roy et al., 2020). Several studies have shown that IMM treatments account for most doses of antimicrobials used on dairy farms (Pol and Ruegg, 2007; González Pereyra et al., 2015; Leite de Campos et al., 2021) but this route comprises a very small proportion of the total mass of antimicrobials sold for use in food animals in the United States. The US Food and Drug Administration (**FDA**) summarizes annual sales of medically important antimicrobials approved for use in cattle, swine, turkeys, and chickens by species, year, and route (FDA, 2023). In 2023, of the >6 million kg of antimicrobials sold for use in food animals, antimicrobials designated for cattle (beef and dairy are reported together) accounted for 41% of the total. However, 93% of the total mass of antimicrobials sold were administered through feed or water. Although sulfadimethoxine can be given orally as a treatment of foot rot, bacterial pneumonia, or shipping fever, administration of medically important antimicrobials to adult dairy cows in the United States in feed or water is prohibited. Most cases of CM and SCM are treated using IMM antimicrobials, which are small, fixed doses as compared with other routes. Of the total antimicrobial mass sold for use in food animals in the United States, <0.01% was labeled for IMM administration, indicating that AMU for treatment of mastitis in dairy cattle accounts for a minute proportion of the total antimicrobials sold for use in food animals.

Reviews of trends in antimicrobial resistance of mastitis pathogens from Canada and the United States have concluded that major mastitis pathogens demonstrate only limited resistance to IMM antimicrobials (Barlow, 2025), although other regions with less restrictive AMU regulations have reported more resistance (Li et al., 2023). A recent systematic review of the public health risks posed by antimicrobial resistant genes (**ARG**) in milk concluded that “considering a low percentage of contaminated milk samples, unknown ARG concentrations, and an unproven role in human disease, the risk attributed to ARG in milk seems to be exaggerated by far. However, the risk of ARG selection on farm, resulting in low treatment success in cattle, is a real one and should be met by prudent use of antibiotics” (Sievers et al., 2025, p. 4508). Although the mass of antimicrobials used to treat mastitis is small, AMU varies considerably among farms, and many farmers can reduce AMU and treatment costs (Leite de Campos et al., 2023, 2024) by modifying mastitis treatment protocols. Enhancing AMS must be focused on encouraging veterinarians and dairy producers to use antimicrobials only when necessary and to use narrow-spectrum antimicrobials when possible. Current and future mastitis treatment protocols will need to emphasize reduced reliance on antimicrobials, especially classes that are critical for the treatment of human diseases.

The current standard of care for CM includes review of severity, etiology, and cow characteristics before prescription of antimicrobials (Ruegg, 2025). Severe cases require immediate therapy, including fluids, anti-inflammatories, and both injectable and IMM antimicrobials. Fortunately, most CM is nonsevere, and many cases achieve spontaneous bacteriological clearance without the need for antimicrobials. When possible, culture-guided therapy should be used to guide treatment of nonsevere CM (de Jong et al., 2023). In most instances, IMM antimicrobials are recommended when CM is caused by gram-positive organisms but are not recommended for CM that is culture-negative nor caused by most gram-negative organisms (Nobrega et al., 2018; Fuenzalida and Ruegg, 2019a). Typical management of cases caused by pathogens with high expectations of spontaneous cure involves monitoring cow health and discarding milk until it returns to a normal appearance. Antimicrobials are not recommended for chronic cases (especially *Staph. aureus*; Barkema et al., 2006) or those that are caused by bacteria outside the spectrum of approved IMM antimicrobials. These cases should be managed to prevent transmission by using segregation, dry-off of chronically affected quarters, or culling. Although researchers have not demonstrated that use of nonsteroidal anti-inflammatory drugs (**NSAID**) consistently improve outcomes of nonsevere CM cases (Latosinski et al., 2020), when the udder is inflamed, NSAID administration should be considered to manage pain. Based on common etiologies, few cases of CM on most dairy farms will benefit from treatment with broad-spectrum IMM antimicrobials (Nobrega et al., 2020). Similarly, a recent review concluded that use of parenteral antimicrobials should be restricted to justifiable cases, such as cows affected with severe mastitis, and that treatment of nonsevere CM should be preferentially performed using noncritically important first-generation cephalosporins rather than critically important antimicrobials such as third-generation cephalosporins (Barlow, 2025). If determination of etiology before treatment is not possible, treatment with a narrow spectrum IMM antimicrobial for the shortest labeled duration is suggested. Cow factors should also be considered before using antimicrobials. Successful elimination of IMI has been associated with age, stage of lactation, negative energy balance, history of previous cases, and environmental factors such as heat stress (Dahl, 2018). With or without antimicrobial therapy, younger cows and cows without a history of subclinical IMI have greater odds of achieving bacteriological clearance and fewer recurrences. Based on principles of AMS, longer-duration therapy cannot be recommended for routine treatments because most cases will not benefit (Ruegg, 2025), and unnecessary AMU is not socially or economically justifiable. When routine treatment protocols exceed labeled minimums for IMM antimicrobials, reducing the duration of treatment should be considered as part of applying AMS on dairy farms.

Several researchers have demonstrated that financial benefits of treatment during lactation are based on balancing the cost of discarded milk with factors that influence the probability of cure (Steeneveld et al., 2007; Steeneveld et al., 2011). Unless waste milk is pasteurized and fed to calves, is rarely cost-effective to treat SCM during lactation, but producers should be aware of potential risks of residues in waste milk to calf health(Pereira et al., 2014). In most instances, both efficacy and economic impact indicate that treatment of SCM is most effective when IMM antimicrobials are given at dry-off.

Administration of IMM antimicrobials in every quarter of each cow that dries off is termed blanket dry cow therapy (**bDCT**). In North America, bDCT became popular in the 1970s due to the high prevalence of quarters affected by SCM. In other regions, selective dry cow therapy (**sDCT**) based on use of IMM antimicrobials only to treat apparently infected quarters was the standard of care (Rajala-Schultz et al., 2021). As the prevalence of SCM has declined in North America, use of sDCT has been promoted (Rowe et al., 2025). Selective dry cow therapy programs promote use of IMM antimicrobials only in cows with existing IMI, and new infections are prevented by use of non-antimicrobial teat sealants. Adoption of sDCT usually results in a 40% to 50% reduction in AMU and in appropriate herds can be used without affecting udder health (McCubbin et al., 2022). Herds that consider sDCT should have bulk tank SCC <250,000 cells/mL and use an appropriate method to identify cows that will benefit from IMM antimicrobials. A simple algorithm that based treatment decisions on occurrence of an individual monthly SCC >200,000 cells/mL or ≥1 case of CM in the ending lactation has been shown to be effective (Rowe et al., 2021). Retrospectively applying this algorithm to records from >35,000 cows in 37 herds, ∼51% of cows would not have required antimicrobial therapy (Leite de Campos et al., 2024), thus indicating considerable potential for reducing AMU. However, based on those criteria, almost 60% of cows in the third or greater lactation would have required antimicrobials, and parity should be considered when using sDCT programs. Use of sDCT is an example of the application of AMS and should be encouraged for herds that meet selection criteria. However, inappropriate herd or cow selection can result in reduced udder health that may require additional antimicrobial treatments (Niemi et al., 2025), so it is important for veterinarians and dairy producers to carefully consider decisions about sDCT on a herd-specific basis.

Mastitis is usually caused by bacteria, and when treatments are indicated for nonsevere cases, antimicrobials remain the drug of choice. Global emphasis on reducing AMU and a desire to provide non-antimicrobial therapies for organic dairy cows has stimulated interest in non-antimicrobial products. Some alternative products (such as anti-inflammatories) do not have antimicrobial activity but may reduce the severity of clinical signs or stimulate immune responses. Other alternatives are novel products with putative bactericidal or bacteriostatic abilities, and the commercial availability of such products varies among countries. Several alternative therapies, including acoustic pulse therapy, quorum sensing boluses, herbal IMM products, and an ozonated food-grade vegetable oil are sold in the United States and have subtle or overt claims for efficacy as mastitis treatments, although none have been FDA approved for this purpose. To date, none of these alternative products have had efficacy documented by independent, controlled clinical trials, and the proposed mechanisms of action suggested for these products remain speculative. Producer testimonials are often provided as evidence of efficacy, but should be viewed cautiously, as pathogen specific outcomes are needed to determine efficacy, and resolution of clinical signs or modest reductions in SCC are not good indicators of achievement of bacteriological clearance.

Identification of efficacious, non-antimicrobial treatments aligns with goals of AMS to reduce AMU on dairy farms. However, a systematic review of currently available alternative treatments was not encouraging (Francoz et al., 2017). Treatments included anti-inflammatories (including flunixin, dexamethasone, prednisone, carprofen, ibuprofen, ketoprofen and others; n = 14 studies), oxytocin (n = 5), biologics (including immunoglobulins and hyperimmune serums; n = 9), homeopathy (n = 5), botanicals (including herbs and aroma therapy; n = 4), probiotics (n = 2) and others (ascorbic acid and riboflavin; n = 2). Bacteriological and clinical cure were the primary outcomes, although the authors noted that most studies had considerable risk of bias and low power to detect differences. Endotoxin challenges were used in most trials that evaluated anti-inflammatories, so results of these studies were focused on alleviation of clinical signs in cows experiencing severe gram-negative mastitis. Some studies did not include either positive or negative controls, and a few did not perform statistical analyses. Although the authors noted that deficiencies in study designs prohibit definitive conclusions for many treatments, they concluded that several treatments (homeopathy, oxytocin with or without frequent milk out, or probiotics) could not be recommended based on lack of evidence of efficacy. The recommendation against probiotics is further supported by a comprehensive review (Rainard and Foucras, 2018) that concluded “there was no sound scientific foundation for the use of probiotics to prevent or treat mastitis” (p. 1). Francoz et al. (2017) noted the urgent need for additional well-designed clinical trials that provide evidence-based recommendations for non-antimicrobial treatments for mastitis.

Several other reviews and numerous research papers have evaluated a variety of potential alternative candidates for treatment of mastitis (Table 2; Rainard and Foucras, 2018; Li et al., 2023; Morales-Ubaldo et al., 2023; Debruyn et al., 2025). Competitive exclusion using organisms such as NAS have been shown to have the potential to inhibit growth of mastitis pathogens (Caddey et al., 2025) and may have potential as a topical treatment to prevent mastitis (Rainard and Foucras, 2018). Unfortunately, most of the proposed treatments have been evaluated only based on their ability to inhibit bacterial growth of selected organisms in vitro and lack evidence of how the compounds behave in vivo or against naturally occurring mastitis cases. The mechanism of action for many of the proposed treatments remains speculative, and additional research is needed to develop practical and effective therapies (Li et al., 2023). Few alternative products have been evaluated using controlled trials and some potential products such as bacteriophages and plant-based products are known to interact with or degrade in milk (Debruyn et al., 2025). Study designs vary among compounds and very few have been adequately powered. Commercial viability of future treatments will require much more rigorous studies that result in sufficient evidence of efficacy to justify costs of the official regulatory processes in various countries. Thus, results of evidence-based studies are needed before the role of alternative therapies for mastitis in AMS programs can be defined.

**Table 2:** Types of alternative therapies for mastitis included in selected published reviews

Compound	Francoz et al., 2017	Li et al., 2023	Morales-Ubaldo et al., 2023	Debruyn et al., 2025
Anti-inflammatories	X	X	X	X
Acoustic pulse therapy		X		X
Bacteriophages		X		
Essential oils	X		X	X
Homeopathy	X		X	X
Nanotechnology		X	X	
Peptides		X	X	X
Phytotherapy	X	X	X	X
Polymers			X	
Probiotics		X		
Others	X	X	X	X

Treatment of mastitis accounts for the majority of AMU on dairy farms. Current and future treatment protocols should be based on knowledge of etiology with the principle of using antimicrobials in a targeted fashion only when indicated. There is considerable opportunity to improve AMS by limiting AMU to cases in which cows will benefit from their use and by using the shortest possible duration of treatment. There is currently little data to support use of most alternative treatments, and resolution of clinical signs should not be used as an indication of efficacy. Use of non-antimicrobial therapies should be supported by scientific evidence that the treatments enhance clinical and bacteriological outcomes in a cost-effective manner. Although treatment of mastitis using antimicrobials is indicated for some cases, bacteriological clearance of many pathogens will occur spontaneously. The development of mastitis results in reduced cow welfare and financial losses. Rather than promoting unproven alternative therapies, our emphasis should be focused on methods to better prevent IMI including development of effective vaccines. Current and future advances in treatment of mastitis should be based on data from well designed, pathogen specific, negatively controlled clinical trials.
